# Identification of a Novel Pathogenic Folliculin Variant in a Chinese Family With Birt–Hogg–Dubé Syndrome (Hornstein-Knickenberg Syndrome)

**DOI:** 10.3389/fgene.2020.565566

**Published:** 2020-11-02

**Authors:** Dandan Zong, Jinhua Li, Xiangming Liu, Ting Guo, Ruoyun Ouyang

**Affiliations:** ^1^Department of Pulmonary and Critical Care Medicine, The Second Xiangya Hospital, Central South University, Changsha, China; ^2^Research Unit of Respiratory Disease, Central South University, Changsha, China; ^3^Diagnosis and Treatment Center of Respiratory Disease, Central South University, Changsha, China

**Keywords:** pneumothorax, folliculin, Birt–Hogg–Dubé syndrome, pulmonary cysts, Hornstein-Knickenberg syndrome, variant

## Abstract

Birt–Hogg–Dubé syndrome (BHDS), which is also called Hornstein-Knickenberg syndrome (HKS), is a hereditary autosomal dominant disorder caused by germline mutations in the folliculin gene (*FLCN*, NM_144997). More pulmonary manifestations (pulmonary cysts and recurrent pneumothoraxes) but fewer skin fibrofolliculomas and renal malignancy are found in Asian BHDS patients compared with other BHDS patients. The atypical manifestation can easily lead to a missed or delayed diagnosis. Here, we report a Chinese family with BHDS that presented with primary spontaneous pneumothorax (PSP) and extensive pulmonary cysts in the absence of skin lesions or renal neoplasms. Next-generation sequencing (NGS) was used to sequence the *FLCN* gene, and Sanger sequencing was carried out on the samples to confirm the presence of these variants. Among the 13 family members, a novel frameshift variant of *FLCN* (c.912delT/p.E305KfsX18) was identified in seven individuals. This variant has not been reported before. Bioinformatics analysis showed that the novel variant might lead to a premature stop codon after 18 amino acid residues in exon 9, and this may affect the expression level of *FLCN*. The identification of this novel frameshift variant of *FLCN* not only further confirms the familial inheritance of BHDS in the proband but also expands the mutational spectrum of the *FLCN* gene in patients with BHDS.

## Introduction

Birt–Hogg–Dubé syndrome (BHDS, OMIM#135150), which is also called Hornstein-Knickenberg syndrome (HKS), is a rare autosomal dominant inherited disorder that predisposes individuals to develop benign skin tumors (fibrofolliculomas), renal neoplasms, and pulmonary cysts with a risk of spontaneous pneumothorax (Menko et al., 2009). This disease was first described by Otto P. Hornstein and Monika Knickenberg as a new autosomal dominant trait characterized by “perifollicular fibromatosis cutis,” multiple skin tags, and multiple colonic polyps with proneness to cancer in 1975 (Hornstein and Knickenberg, 1975). Two years later, Arthur R. Birt, Georgina R. Hogg, and W. James Dube (Birt and Hogg, 1977) from Canada described similar hereditary skin lesions, fibrofolliculoma, without any extracutaneous cancer proneness. Then, the disease was named as “BHDS” after the three Canadian doctors. Today, many authors believe that “fibrofolliculoma” is identical with “perifollicular fibroma” (Happle, 2020). Thus, the two diseases are essentially the same. Today, BHDS is more widely accepted, but we should not forget the persons who first discovered the disease.

The most common skin lesions of BHDS are cutaneous fibrofolliculomas occurring on the head, neck, and upper body of greater than 85% of BHDS-affected individuals over 25 years of age (Schmidt and Linehan, 2018). Renal tumors occur frequently in BHDS patients, and the most frequent histological types of neoplasms are hybrid oncocytic/chromophobe tumors or chromophobe renal cell carcinoma (Furuya et al., 2020). More than 80% of BHDS patients have multiple bilateral lung cysts and approximately 22.5–38% patients report a history of single or recurrent spontaneous pneumothorax, which can present as the first symptom (Sattler et al., 2020). The CT examination results showed that the cysts in the lungs were variable with well-defined walls, located mostly in the basal medial regions (58%), less frequently in the basal peripheral regions (27%), and approximately 40% adhere to the pleura. The number of cysts varies from tens to hundreds, and their size also varies greatly, from a few millimeter to 2 cm or more, but most have a diameter smaller than 1 cm (Tobino et al., 2011). Spontaneous pneumothorax may be the only manifestation in BHDS patients, which has been described in many reports (Graham et al., 2005; Painter et al., 2005). It is often misdiagnosed as primary spontaneous pneumothorax (PSP), in particular, in the cases with only isolated lung cysts/pneumothorax presentation (Min et al., 2020). Lack of a comprehensive and profound understanding of BHDS often leads to a misdiagnosis.

The folliculin (*FLCN*) gene is considered to be a tumor suppressor. Localized in the short arm of chromosome 17p11.2, it contains 14 exons, 11 of which encode a 579-amino-acid-long protein called folliculin (Han et al., 2020). Germline mutations of the *FLCN* gene were identified to be responsible for BHDS in 2002 (Nickerson et al., 2002). *FLCN* is expressed in normal skin cells, nephrons, stromal cells, Type I pneumocytes, and the acinar cells of the pancreas and parotid gland. Pathogenic *FLCN* variants may lead to the inactivation of the gene, which destroys the ability of *FLCN* to restrict cell growth and division, resulting in deregulated cell growth and protein synthesis, giving rise to the formation of malignant and benign tumors (Balsamo et al., 2020). To date, more than 280 different types of unique mutations spanning the entire coding region of the *FLCN* gene have been identified, according to the Leiden Open Variation Database.^1^ The majority of *FLCN* mutations identified in the germline of BHDS patients are frameshifts (insertion/deletion), nonsense mutations, and splice site mutations (Lim et al., 2010). It has been observed in BHDS patients that *FLCN* mutations in exon 9 are associated with an increased number of lung cysts and exon 9 and 12 mutations are correlated with more episodes of spontaneous pneumothorax (Toro et al., 2007). These genotype-phenotype correlations have only been suggested in some studies.

Here, we reported a large Chinese family in which seven members from three generations developed lung cysts. A genetic study revealed a novel and previously not reported variant in *FLCN* in the seven individuals.

## Materials and Methods

### Patient Characteristics

A 43-year-old female proband from Central China was diagnosed with PSP in the Second Xiangya Hospital of the Central South University. The family histories of her 12 relatives from three generations were collected. Written informed consent was obtained from the individuals for the publication of any potentially identifiable images or data included in this article. All subjects received skin examination by professional dermatologists. Chest CT testing was done to evaluate the pulmonary lesion. And abdominal ultrasound examination was performed to rule out renal involvement. Blood was collected from the probands and their family members.

### DNA Extraction

Peripheral blood samples were collected into EDTA anticoagulant tubes and stored at +4°C until DNA isolation was performed within 24 h of collection. Genomic DNA was prepared using a DNeasy Blood & Tissue Kit (Qiagen, Valencia, CA, United States) according to the instructions of the manufacturer. The DNA samples were stored at −20°C until the PCR stage.

### Mutation Sequencing

The entire coding regions, including the intronic flanking sequences of FLCN were amplified by PCR. The software IDT^2^ was used to design primers for PCR (primer sequences will be provided upon requests). Sequences of PCR products were determined by the ABI 3100 Genetic Analyzer (Thermo Fisher Scientifc, Inc., Waltham, MA, United States). The DNA sequencing reaction of the proband was performed using the next-generation sequencing (NGS) method according to manufacturer’s protocols (details are available upon request). The multiple FLCN protein sequences were aligned using the program MUSCLE (version 3.6). The online databases, PolyPhen-2 (polymorphism phenotyping), and MutationTaster programs were used to predict the possible effects of variants on the function of the proteins.

### Sanger Sequencing

To validate true positive novel variants identified by NGS, Sanger sequencing was carried out on the samples to confirm the presence or absence of these variants in the proband, and other family members. The sequencing results were later analyzed using Sequencher software (Gene Codes Corporation, MI, United States).

## Results

### Germline Variant of the *FLCN* Gene

**Figure 1:**
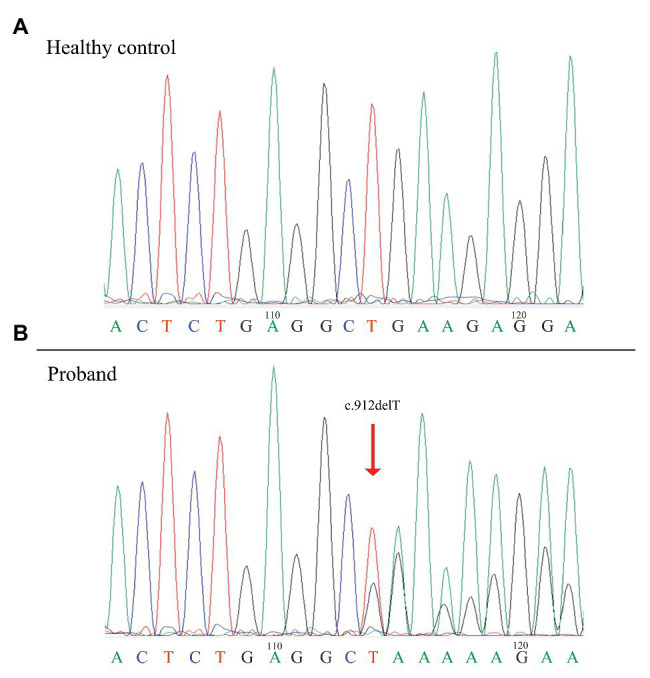
Identification of a novel frameshift mutation of the *FLCN* gene in the patients. Sanger sequencing showing the novel *FLCN* mutation in exon 9 of the proband compared with a healthy control. The red arrow indicates the site of the mutation (c.912delT/p.E305KfsX18). *FLCN*: Folliculin.

**Figure 2:**
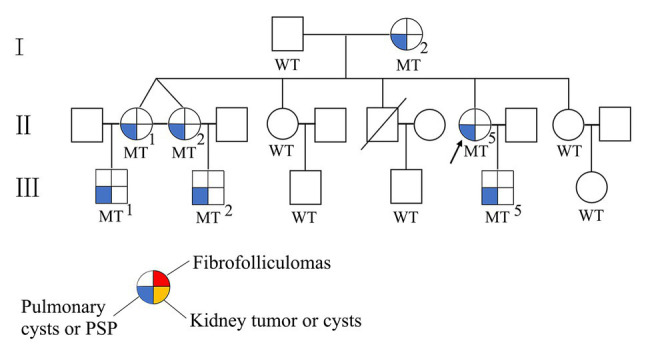
The pedigree of the Chinese family with the *FLCN* mutation. Squares indicate male family members; and circles indicate female members. MT, mutant; WT, wildtype; arrow indicates proband.

Sequence analysis of the *FLCN* gene revealed a novel deletion variant (c.912delT/p.E305KfsX18) in exon 9. This variant was then confirmed by Sanger sequencing (Figure 1). The c.912delT variant resulted in a frameshift at amino acid position 305 and the introduction of a premature stop codon after 18 amino acid residues (p.E305KfsX18) and was predicted to be disease-causing by MutationTaster.^3^ In total, seven of the 13 family members harbored the same variant. The pedigree of the family members included in the study is shown in Figure 2. Among the affected individuals, four were female, and three were male. No variant at this site was found in any of the available unaffected family members. The newly identified variant has never been reported in previous studies. According to the dbSNP and Human Gene Mutation Database,^4^ this heterozygous variant is novel.

### Clinical Characteristics

**Figure 3:**
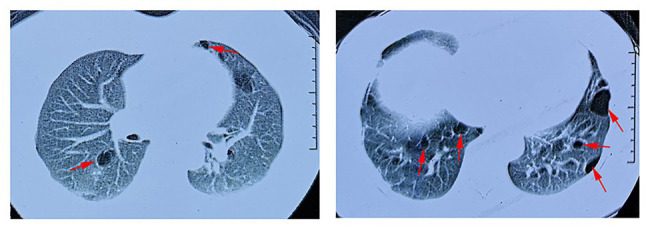
Lung CT scan of the proband. Red arrows indicate multiple pulmonary cysts.

In our study, all seven patients with the *FLCN* variant showed bilateral multiple pulmonary cysts on CT imaging (Figure 3). PSP was detected in three cases, including the proband. Two of the family members underwent conservative treatment due to PSP, while the proband received thoracoscopic bullectomy for a first episode of right-lung pneumothorax. The pathological section of her lung pathology presents a pulmonary cyst. None of the patients exhibited fibrofolliculomas skin lesions or renal involvement. The two family members who suffered from PSP were misdiagnosed with pulmonary cysts due to the atypical manifestations, and they did not receive *FLCN* gene mutation screening. The other four patients without pneumothorax underwent chest CT, and pulmonary cysts were observed for the first time in the present study. The clinical characteristics of most of the living family members are summarized in Table 1.

**Table 1:** Clinical features of family members with folliculin (FLCN) gene mutation.

Patient No.	Sex	Age	BMI (kg/m^2^)	Smoking history	Age at first episode of pneumothorax	No. of PSP attack	Treatment	Lung cysts	Skin lesions	Kidney lesions
I-2	F	75	21.2	N	56	1	TD	Bilateral, multiple	N	N
II-1	F	54	23.1	N	N	0	N	Bilateral, multiple	N	N
II-2	F	54	24.5	N	49	1	TD	Bilateral, multiple	N	N
II-5	F	44	22.8	N	43	1	VB + MP	Bilateral, multiple	N	N
III-1	M	29	25.6	Y	N	0	N	Bilateral, multiple	N	N
III-2	M	31	24.9	Y	N	0	N	Bilateral, multiple	N	N
III-5	M	20	23.7	N	N	0	N	Bilateral, multiple	N	N

BMI, body mass index; F, female; M, male; Y, yes; N, no; TD, tube drainage; VB, video-assisted thoracoscopic surgery bullectomy; MP, mechanical pleurodesis.

## Discussion

In this study, we presented a novel frameshift variant (c.912delT/p.E305KfsX18) in exon 9 of the *FLCN* gene, which has not been previously reported in individuals with BHDS. Frameshift mutation may lead to an early termination of protein synthesis or to nonsense-mediated mRNA decay in which the defective mRNA is prematurely degraded (Ali et al., 2020).

The typical clinical manifestations of BHDS are skin, kidney, and lung involvement. However, patients do not always have all the three characteristic manifestations. Toro et al. (2008) reported that most Caucasians with BHDS (85–90%) have skin lesions as their major complaint, 34% of patients developed kidney tumors, and the incidence of pneumothorax is about one-third. A Japanese study conducted by Furuya et al. (2016) found that recurrent episodes of pneumothorax were more prevalent (73.7%) in BHDS patients and were more informative as diagnostic criteria for BHDS in their Japanese Asian population. In contrast, cutaneous manifestations are not the major complaint in the Japanese population (Furuya et al., 2016). BHDS patients in China also present with more pulmonary manifestations but fewer skin lesions and renal malignancies (Ren et al., 2008; Liu et al., 2017). Selection bias may be responsible for the different frequencies of pulmonary manifestations between Asian and Western BHDS cases (Liu et al., 2019). Most Caucasian patients with BHDS were recruited through referrals from departments of dermatology or urology, while most patients with BHDS in China were diagnosed by respiratory physicians due to the pulmonary manifestations (Liu et al., 2019). Patients with cutaneous fibrofolliculoma or renal neoplasms may not receive regular chest CT examinations if the patients do not have any marked respiratory symptoms. Apart from renal tumors, several other tumor entities have been reported in association with BHDS, including colon polyps and tumors, breast cancer, lung cancer, thyroid cancer, parathyroid adenoma, lipoma, melanoma, and parotid oncocytoma (Steinlein et al., 2018). Among these tumors, colon cancer has been investigated in more detail. However, the incidence of colon cancer varies greatly among different studies (Zbar et al., 2002; Nahorski et al., 2010; Steinlein et al., 2018). In Asian, there are only sporadic reports about BHDS accompanied by colon polyposis or carcinoma (Kashiwada et al., 2012; Motegi et al., 2018). Whether these tumors are genuine associations or merely coincidences with BHDS has not yet been clinically validated.

In the present study, the proband and her six relatives also exhibited pulmonary involvement without skin or renal lesions, and three out of seven (42.8%) members suffered from PSP. These manifestations are in accordance with the previous studies in China (Ren et al., 2008; Liu et al., 2017). Since renal neoplasms frequently occur in BHDS patients, surveillance by renal imaging annually, such as low dose spiral CT or MRI (Hindman, 2018; Kay and Pedrosa, 2018), should be carried out in this family to identify any kidney tumors as early as possible. Due to the nontypical clinical manifestations, it is difficult to diagnose BHDS in patients without cutaneous lesions or renal pathology. *FLCN* mutation screening is recommended and is a reliable method for the clinical molecular diagnosis of BHDS, especially in patients who do not have skin and renal manifestations (Zheng et al., 2019).

The *FLCN* gene encodes the protein folliculin, a 579-amino-acid-long protein, which was first reported to be responsible for BHDS in 2002 (Nickerson et al., 2002). Since then, over 280 *FLCN* gene mutations have been identified according to the Leiden Open Variation Database. Half of BHDS families exhibited frameshift mutations in coding exons of *FLCN* (Furuya et al., 2016), while splice site, nonsense, missense, and deletion mutations are less prevalent (Kim et al., 2012).

The genotype-phenotype correlations between *FLCN* mutation status and skin, lung, or renal manifestations are still not clear. Some researchers have reported that *FLCN* mutations in exon 9 and 12 are associated with a higher number of pulmonary cysts, a larger cyst diameter, and more episodes of pneumothorax (Toro et al., 2007). A deleted cytosine in exon 11 results in a significantly lower frequency of renal neoplasia compared with the patients with an inserted cytosine at the same location (Schmidt et al., 2005). In the present study, a heterozygous frameshift variant (c.912delT/p.E305KfsX18) was detected in exon 9 of *FLCN* of seven members from three generations of the same family. This novel variant is predicted to cause premature truncation of the *FLCN* protein, leading to functional haploinsufficiency of *FLCN*. Loss of function of this protein can cause alveolar enlargement and cysts formation, consequently leading to pneumothorax (Xing et al., 2017).

The exact mechanism of *FLCN* mutations leading to pulmonary cysts and pneumothorax in BHDS patients has not yet been fully elucidated. The stretch hypothesis has been proposed based on observations of increased cell-cell adhesion in *FLCN*-deficient cells, which may reduce the flexibility of the cell-cell junctions, resulting in stretch-induced lung injury and subsequent airspace enlargement (Medvetz et al., 2012; Kennedy et al., 2016). Animal studies have shown that *FLCN* deletion in SP-C expressing lung epithelial cells leads to alveolar enlargement and impaired lung function by inducing alveolar epithelial cell apoptosis (Goncharova et al., 2014). A recent study (Chu et al., 2020) showed that the deletion of mesenchymal *FLCN* resulted in a reduction of postnatal alveolar formation and destruction of alveolar walls through the suppression of cell proliferation and alveolar myofibroblast differentiation, and the inhibition of extracellular matrix proteins and elastin expression. These results suggested that *FLCN* deficiency may lead to pulmonary cystic lesions and pneumothorax by inducing alveolar hypoplasia. So far, most of the studies have concentrated on the functions of *FLCN* outside the lung, so the exact mechanism of how the *FLCN* gene and involved pathways contribute to lung cyst formation is very limited.

## Conclusion

In conclusion, we found a novel heterozygous frameshift variant in exon 9 of *FLCN* (c.912delT/p.E305KfsX18) in a Chinese BHDS family, which might cause lung cysts and PSP. These findings highlight the importance of screening for *FLCN* gene variants in patients with pulmonary cysts and PSP, even in the absence of skin and/or kidney lesions. The identification of this novel variant expands the mutation spectrum of the *FLCN* gene in the Chinese population.

## Data Availability Statement

All datasets generated for this study are included in the article, further inquiries can be directed to the corresponding author.

## Ethics Statement

The studies involving human participants were reviewed and approved by the Review Board of The Second Xiangya Hospital of the Central South University. The patients/participants provided their written informed consent obtained from the individuals for the publication of any potentially identifiable images or data included in this article.

## Author Contributions

All authors contributed to the reviewing of the paper. DZ and XL performed the laboratory work, statistical analyses, and drafted the manuscript. JL and RO collected the clinical data. TG performed NGS analysis. RO supervised the study and helped to revise the manuscript. All authors contributed to the article and approved the submitted version.

### Conflict of Interest

The authors declare that the research was conducted in the absence of any commercial or financial relationships that could be construed as a potential conflict of interest.

## Footnotes

^1^https://www.lovd.nl/

^2^http://www.idtdna.com/site

^3^www.mutationtaster.org

^4^http://www.hgmd.cf.ac.uk/ac/index.php

## References

[ref1] Ali M. Z. Blatterer J. Khan M. A. Schaflinger E. Petek E. Ahmad S.. (2020). Identification of a novel protein truncating mutation p.Asp98* in XPC associated with xeroderma pigmentosum in a consanguineous Pakistani family. Mol. Genet. Genomic Med. 8:e1060. 10.1002/mgg3.1060, PMID: 31923348 PMC7005610

[ref2] Balsamo F. Cardoso P. A. S. do Amaral Junior S. A. Theodoro T. R. de Sousa Gehrke F. da Silva Pinhal M. A.. (2020). Birt-Hogg-Dube syndrome with simultaneous hyperplastic polyposis of the gastrointestinal tract: case report and review of the literature. BMC Med. Genet. 21:52. 10.1186/s12881-020-0991-8, PMID: 32171268 PMC7071710

[ref3] Birt A. R. Hogg G. R. Dube W. J. (1977). Hereditary multiple fibrofolliculomas with trichodiscomas and acrochordons. Arch. Dermatol. 113, 1674–1677. 10.1001/archderm.1977.01640120042005, PMID: 596896

[ref4] Chu L. Luo Y. Chen H. Miao Q. Wang L. Moats R.. (2020). Mesenchymal folliculin is required for alveolar development: implications for cystic lung disease in Birt-Hogg-Dube syndrome. Thorax 75, 486–493. 10.1136/thoraxjnl-2019-214112, PMID: 32238524

[ref5] Furuya M. Hasumi H. Yao M. Nagashima Y. (2020). Birt-Hogg-Dube syndrome-associated renal cell carcinoma: histopathological features and diagnostic conundrum. Cancer Sci. 111, 15–22. 10.1111/cas.14255, PMID: 31777168 PMC6942440

[ref6] Furuya M. Yao M. Tanaka R. Nagashima Y. Kuroda N. Hasumi H.. (2016). Genetic, epidemiologic and clinicopathologic studies of Japanese Asian patients with Birt-Hogg-Dube syndrome. Clin. Genet. 90, 403–412. 10.1111/cge.12807, PMID: 27220747

[ref7] Goncharova E. A. Goncharov D. A. James M. L. Atochina-Vasserman E. N. Stepanova V. Hong S. B.. (2014). Folliculin controls lung alveolar enlargement and epithelial cell survival through E-cadherin, LKB1, and AMPK. Cell Rep. 7, 412–423. 10.1016/j.celrep.2014.03.025, PMID: 24726356 PMC4034569

[ref8] Graham R. B. Nolasco M. Peterlin B. Garcia C. K. (2005). Nonsense mutations in folliculin presenting as isolated familial spontaneous pneumothorax in adults. Am. J. Respir. Crit. Care Med. 172, 39–44. 10.1164/rccm.200501-143OC, PMID: 15805188

[ref9] Han J. Hao J. Liu R. Xie Y. Kang Z. (2020). Birt-Hogg-Dube syndrome caused by a mutation of FLCN gene in a CVST patient: a case report. Int. J. Neurosci. 130, 438–442. 10.1080/00207454.2019.1691204, PMID: 31694440

[ref37] Happle R. (2020). Hornstein-Knickenberg syndrome vs. Birt-Hogg-Dubé syndrome: a critical review of an unjustified eponymic designation. J. Eur. Acad. Dermatol. Venereol. 34, 885–887. 10.1111/jdv.16190 31923324

[ref10] Hindman N. M. (2018). Imaging of cystic renal masses. Urol. Clin. North Am. 45, 331–349. 10.1016/j.ucl.2018.03.006, PMID: 30031458

[ref11] Hornstein O. P. Knickenberg M. (1975). Perifollicular fibromatosis cutis with polyps of the colon--a cutaneo-intestinal syndrome sui generis. Arch. Dermatol. Res. 253, 161–175. 10.1007/BF00582068, PMID: 1200700

[ref12] Kashiwada T. Shimizu H. Tamura K. Seyama K. Horie Y. Mizoo A. (2012). Birt-Hogg-Dube syndrome and familial adenomatous polyposis: an association or a coincidence? Intern. Med. 51, 1789–1792. 10.2169/internalmedicine.51.7239, PMID: 22790147

[ref13] Kay F. U. Pedrosa I. (2018). Imaging of solid renal masses. Urol. Clin. North Am. 45, 311–330. 10.1016/j.ucl.2018.03.013, PMID: 30031457 PMC6057157

[ref14] Kennedy J. C. Khabibullin D. Henske E. P. (2016). Mechanisms of pulmonary cyst pathogenesis in Birt-Hogg-Dube syndrome: the stretch hypothesis. Semin. Cell Dev. Biol. 52, 47–52. 10.1016/j.semcdb.2016.02.014, PMID: 26877139

[ref15] Kim J. Yoo J. H. Kang D. Y. Cho N. J. Lee K. A. (2012). Novel in-frame deletion mutation in FLCN gene in a Korean family with recurrent primary spontaneous pneumothorax. Gene 499, 339–342. 10.1016/j.gene.2012.03.037, PMID: 22446046

[ref16] Lim D. H. Rehal P. K. Nahorski M. S. Macdonald F. Claessens T. Van Geel M.. (2010). A new locus-specific database (LSDB) for mutations in the folliculin (FLCN) gene. Hum. Mutat. 31, E1043–E1051. 10.1002/humu.21130, PMID: 19802896

[ref17] Liu Y. Xu Z. Feng R. Zhan Y. Wang J. Li G.. (2017). Clinical and genetic characteristics of chinese patients with Birt-Hogg-Dube syndrome. Orphanet J. Rare Dis. 12:104. 10.1186/s13023-017-0656-7, PMID: 28558743 PMC5450333

[ref18] Liu K. Xu W. Tian X. Xiao M. Zhao X. Zhang Q.. (2019). Genotypic characteristics of Chinese patients with BHD syndrome and functional analysis of FLCN variants. Orphanet J. Rare Dis. 14:223. 10.1186/s13023-019-1198-y, PMID: 31615547 PMC6794894

[ref19] Medvetz D. A. Khabibullin D. Hariharan V. Ongusaha P. P. Goncharova E. A. Schlechter T.. (2012). Folliculin, the product of the Birt-Hogg-Dube tumor suppressor gene, interacts with the adherens junction protein p0071 to regulate cell-cell adhesion. PLoS One 7:e47842. 10.1371/journal.pone.0047842, PMID: 23139756 PMC3490959

[ref20] Menko F. H. van Steensel M. A. Giraud S. Friis-Hansen L. Richard S. Ungari S.. (2009). Birt-Hogg-Dube syndrome: diagnosis and management. Lancet Oncol. 10, 1199–1206. 10.1016/S1470-2045(09)70188-3, PMID: 19959076

[ref21] Min H. Ma D. Zou W. Wu Y. Ding Y. Zhu C.. (2020). FLCN-regulated miRNAs suppressed reparative response in cells and pulmonary lesions of Birt-Hogg-Dube syndrome. Thorax 75, 476–485. 10.1136/thoraxjnl-2019-213225, PMID: 32184379 PMC7279199

[ref22] Motegi S. I. Sekiguchi A. Fujiwara C. Yamazaki S. Nakano H. Sawamura D.. (2018). A case of Birt-Hogg-Dube syndrome accompanied by colon polyposis and oral papillomatosis. Eur. J. Dermatol. 28, 720–721. 10.1684/ejd.2018.3394, PMID: 30325331

[ref23] Nahorski M. S. Lim D. H. Martin L. Gille J. J. McKay K. Rehal P. K.. (2010). Investigation of the Birt-Hogg-Dube tumour suppressor gene (FLCN) in familial and sporadic colorectal cancer. J. Med. Genet. 47, 385–390. 10.1136/jmg.2009.073304, PMID: 20522427

[ref24] Nickerson M. L. Warren M. B. Toro J. R. Matrosova V. Glenn G. Turner M. L.. (2002). Mutations in a novel gene lead to kidney tumors, lung wall defects, and benign tumors of the hair follicle in patients with the Birt-Hogg-Dube syndrome. Cancer Cell 2, 157–164. 10.1016/s1535-6108(02)00104-6, PMID: 12204536

[ref25] Painter J. N. Tapanainen H. Somer M. Tukiainen P. Aittomaki K. (2005). A 4-bp deletion in the Birt-Hogg-Dube gene (FLCN) causes dominantly inherited spontaneous pneumothorax. Am. J. Hum. Genet. 76, 522–527. 10.1086/428455, PMID: 15657874 PMC1196403

[ref26] Ren H. Z. Zhu C. C. Yang C. Chen S. L. Xie J. Hou Y. Y.. (2008). Mutation analysis of the FLCN gene in Chinese patients with sporadic and familial isolated primary spontaneous pneumothorax. Clin. Genet. 74, 178–183. 10.1111/j.1399-0004.2008.01030.x, PMID: 18505456

[ref27] Sattler E. C. Syunyaeva Z. Mansmann U. Steinlein O. K. (2020). Genetic risk factors for spontaneous pneumothorax in Birt-Hogg-Dube syndrome. Chest 157, 1199–1206. 10.1016/j.chest.2019.12.019, PMID: 31958439

[ref28] Schmidt L. S. Linehan W. M. (2018). FLCN: the causative gene for Birt-Hogg-Dube syndrome. Gene 640, 28–42. 10.1016/j.gene.2017.09.044, PMID: 28970150 PMC5682220

[ref29] Schmidt L. S. Nickerson M. L. Warren M. B. Glenn G. M. Toro J. R. Merino M. J.. (2005). Germline BHD-mutation spectrum and phenotype analysis of a large cohort of families with Birt-Hogg-Dube syndrome. Am. J. Hum. Genet. 76, 1023–1033. 10.1086/430842, PMID: 15852235 PMC1196440

[ref30] Steinlein O. K. Ertl-Wagner B. Ruzicka T. Sattler E. C. (2018). Birt-Hogg-Dube syndrome: an underdiagnosed genetic tumor syndrome. J. Dtsch. Dermatol. Ges. 16, 278–283. 10.1111/ddg.13457, PMID: 29537177

[ref31] Tobino K. Gunji Y. Kurihara M. Kunogi M. Koike K. Tomiyama N.. (2011). Characteristics of pulmonary cysts in Birt-Hogg-Dube syndrome: thin-section CT findings of the chest in 12 patients. Eur. J. Radiol. 77, 403–409. 10.1016/j.ejrad.2009.09.004, PMID: 19782489

[ref32] Toro J. R. Pautler S. E. Stewart L. Glenn G. M. Weinreich M. Toure O.. (2007). Lung cysts, spontaneous pneumothorax, and genetic associations in 89 families with Birt-Hogg-Dube syndrome. Am. J. Respir. Crit. Care Med. 175, 1044–1053. 10.1164/rccm.200610-1483OC, PMID: 17322109 PMC1899269

[ref33] Toro J. R. Wei M. H. Glenn G. M. Weinreich M. Toure O. Vocke C.. (2008). BHD mutations, clinical and molecular genetic investigations of Birt-Hogg-Dube syndrome: a new series of 50 families and a review of published reports. J. Med. Genet. 45, 321–331. 10.1136/jmg.2007.054304, PMID: 18234728 PMC2564862

[ref34] Xing H. Liu Y. Jiang G. Li X. Hou Y. Yang F.. (2017). Clinical and genetic study of a large Chinese family presented with familial spontaneous pneumothorax. J. Thorac. Dis. 9, 1967–1972. 10.21037/jtd.2017.06.69, PMID: 28839995 PMC5542951

[ref35] Zbar B. Alvord W. G. Glenn G. Turner M. Pavlovich C. P. Schmidt L.. (2002). Risk of renal and colonic neoplasms and spontaneous pneumothorax in the Birt-Hogg-Dube syndrome. Cancer Epidemiol. Biomark. Prev. 11, 393–400. PMID: 11927500

[ref36] Zheng C. M. Hu X. X. Gao Y. L. Miao J. B. Li H. (2019). Recurrent primary spontaneous pneumothorax in a large Chinese family: a clinical and genetic investigation. Chin. Med. J. 132, 2402–2407. 10.1097/CM9.0000000000000442, PMID: 31567476 PMC6831060

